# Eosinophilic Esophagitis in a 75-Year-Old Saudi Man: A Case Report

**DOI:** 10.7759/cureus.34705

**Published:** 2023-02-06

**Authors:** Saad S Alkhowaiter

**Affiliations:** 1 Medicine/Gastroenterology, College of Medicine, King Saud University, King Khalid University Hospital, Riyadh, SAU

**Keywords:** gastroesophageal reﬂux disease, proton pump inhibitor, elderly, case report, eosinophilic esophagitis

## Abstract

The incidence and prevalence of eosinophilic esophagitis (EoE) are increasing in adults and children worldwide. Once thought primarily to affect children and young adults, EoE is now recognized in all age groups. To our knowledge, this case study is the first known report of EoE diagnosed in an elderly man in Saudi Arabia. The 75-year-old patient presented with a chief complaint of dry mouth and mild heartburn symptoms. Further history inquiry disclosed that he experienced dysphagia occasionally. His endoscopy findings revealed signs associated with EoE, which was confirmed by biopsy showing marked infiltration of eosinophils (>30/hpf) in upper and lower esophagus. Following treatment with dexlansoprazole for eight weeks, the patient reported no further symptoms, and he remained in remission three months thereafter. Elderly patients with EoE may display atypical signs and symptoms and rarely have concomitant allergy; EoE should be considered in older patients especially those with dysphagia.

## Introduction

Eosinophilic esophagitis (EoE) is being increasingly acknowledged as a common cause of upper gastrointestinal (GI) morbidity in both children and adults [1-3]. First described as a distinct esophageal disease in the mid-1990s, emerging evidence suggests that EoE is more widespread than originally believed [4]. A recent study conducted in the Danish general population reported the prevalence of EoE as 69.7 per 100,000 people [5]. Traditionally, EoE has been considered predominately to affect males, children, and young adults [3,6]. Indeed, EoE is most often seen in men aged between 20 and 50 years [1]. Cases occurring in older age groups are rare [7].

Although natural history data are limited, it is well established from observations in prospective and retrospective cohort studies that EoE is a chronic disease that in many cases, especially in adults, can be symptomatic for several years or decades prior to diagnosis [1]. It is also believed that many patients progress from an inflammatory to a fibrotic process over time, which might explain the differences in clinical presentation in children versus adults with EoE [1]. The objective of this case presentation was to document the occurrence of EoE in an elderly patient in Saudi Arabia, which, to our knowledge, hasn't been reported before in Saudi Arabia.

## Case presentation

A 75-year-old Saudi man visited my gastroenterology clinic (September 2022) complaining of persistent dry mouth with daily mild heartburn for the past one year. He did not voluntarily report dysphagia with meals. However, further history inquiry revealed he experienced mild intermittent solid food dysphagia with no weight loss. He did not have any other significant gastrointestinal symptoms. The patient emphasized he was seeking medical attention for dry mouth. His physical examination was unremarkable. Hematological and biochemical parameters were within normal limits. He was otherwise healthy had not taken any recent medications.

**Figure 1:**
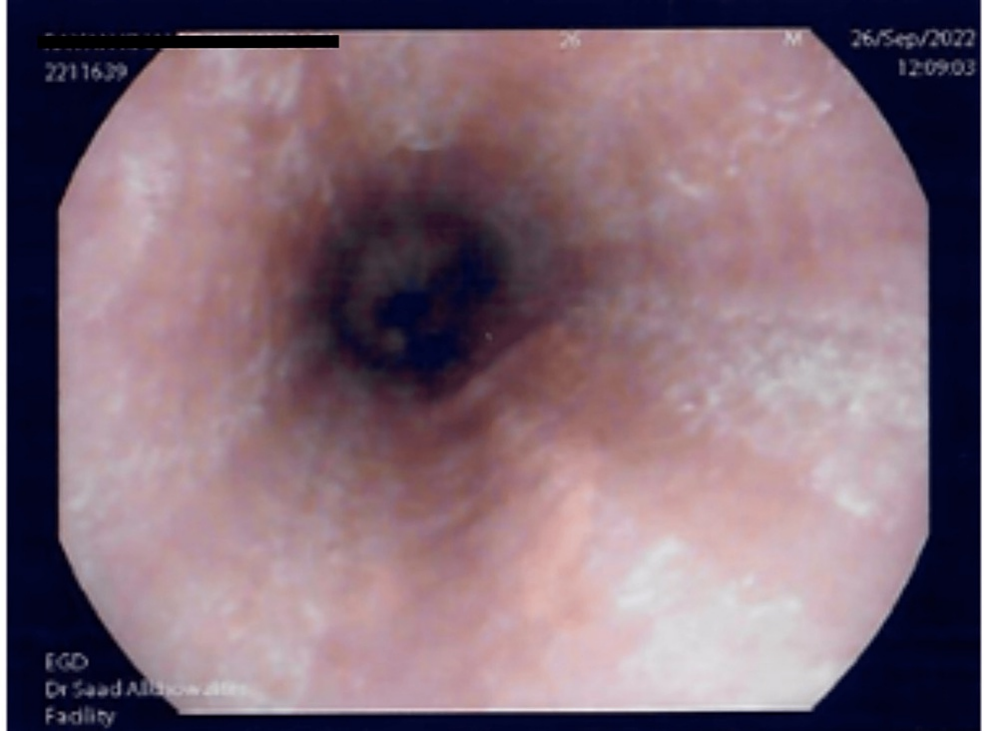
Upper endoscopy image of the patient’s esophageal appearance on initial presentation. Mild furrows and whitish exudates are among the typical visible features in the esophageal lumen of patients with eosinophilic esophagitis

The upper endoscopy examination showed Los Angeles (LA) grade A esophagitis with some patches of whitish exudates in the distal esophagus (Figure 1) [8]. Biopsy specimens were obtained from the proximal and distal esophagus (three samples per segment); a histologic examination (by the in-hospital pathologist with an interest in gastroenterology) revealed marked infiltration of more than 30 eosinophils/optical high power field (hpf) in the proximal and distal esophagus, confirming the diagnosis of EoE.

The patient was prescribed dexlansoprazole 30 mg twice daily before meals for eight weeks, and achieved excellent clinical response in terms of resolution of all symptoms including dry mouth. Repeat endoscopy performed one week after the completion of eight-week proton pump inhibitor (PPI) therapy showed a normal esophagus. His esophageal biopsies suggested histologic remission (cell count, 1-3 eosinophils/hpf). The patient remained symptom-free at the three-month post-treatment follow-up.

## Discussion

EoE is known as a clinicopathologic condition characterized by dysphagia related to food allergy, incriminating endoscopic particulars, and treatment response to topical steroids, PPIs, or elimination diets [1]. The term clinicopathologic recognizes that none of the clinical attributes or endoscopic appearance of EoE is pathognomonic when considered in isolation [1]. Rather, the diagnosis is confirmed in a patient presenting with EoE symptoms whose endoscopy-guided esophageal squamous epithelial biopsy results demonstrate eosinophilic infiltration with a cell count >15 eosinophils/hpf [1,3].

The number of patients with EoE has been found to be increasing. Affecting people of all ages, several studies consistently show the condition is more prevalent in adults compared to children [9,10]. The average patient presenting with EoE is aged 20 to 50 years. Cases occurring in older age are rarely seen [7]. Elderly patients with EoE may be missed possibly because EoE has been traditionally believed to mainly affect younger people, and physicians are unlikely to seek the diagnosis in older-aged people, especially if they exhibit symptoms of gastroesophageal reflux disease (GERD) only and respond to PPIs. Or, elderly patients may present with atypical symptoms and in them EoE might be overlooked [7].

In a broad US population-based study using data from a national pathology database, among 74,162 unique patients who provided upper GI biopsies, a total of 363 patients (mean age, 37.6, range 1-98, years) were identified as EoE cases [6]. Among these 363 cases, the largest proportion was found in the 30- to 40-year age range; 42 (12%) cases were children aged under 18 years whereas 25 (<7%) were aged between 60 and 69 years and only 14 (<4%) cases were over 70 years old [6].

In the Netherlands, a nationwide register-based pathology search spanning 25 years (1995-2019) identified 4061 patients with a confirmed diagnosis of EoE. The mean age at diagnosis was 37.9 years, and most patients (54%) were aged between 20 and 49 years whereas 12% were aged 60-79; 1% were over 80 years of age [11]. In a similar fashion, Maradey-Romero’s group analyzed EoE-related data aggregated from electronic health records (EHRs) of nearly 15 million patients receiving treatment at US institutions participating in the Explorys private cloud-based platform searchable gateway (Explorys Inc., Cleveland, OH) [7]. In the population studied, the overall prevalence of EoE was 50.6 per 100,000 patients (0.05%). Among elderly individuals aged over 65 years, the EoE prevalence was 18.6 per 100,000 patients (0.01%), a significantly lower rate versus younger patients (p<0.001). Elderly patients were also significantly less likely to report symptoms such as heartburn, nausea, and abdominal pain or to have food allergies than younger individuals. On the other hand, a significantly higher proportion of elderly than younger adult EoE patients had a concomitant diagnosis of GERD (p<0.001) [7].

These epidemiology studies are remindful that despite the relatively low numbers of elderly patients with EoE, there remains a need to consider EoE in older patients who present with typical clinical symptoms, especially dysphagia.

Proposed explanations for the observation that EoE is less commonly encountered in older than in younger adults have produced some intriguing possibilities [2]. These include better general hygiene practices adopted starting from the later half of the 20th century that may have led to widespread lowered human immune tolerance and thereby provoked the pathogenesis of allergic diseases including EoE in children born more recently than those who comprise the current elderly population. Other researchers have pointed out a negative inverse association between EoE and *Helicobacter pylori* infection; since this bacterial organism was first detected and largely eradicated by antibiotics in the early 1980s, lack of *H. pylori* in people born since that time might explain the higher prevalence of EoE observed in subsequent generations compared with older people. Although these two proposed explanations for the observed trend of a higher prevalence of EoE in younger versus older adults are interesting, there are no direct data to support either contention [2].

The present patient’s main clinical complaint was dry mouth. Dry mouth is not a typical sign of EoE; however, during consultation, he disclosed a history of frequent heartburn accompanied by intermittent dysphagia. Dysphagia and heartburn are commonly seen in patients with not only EoE but also GERD especially if complicated with stricture. Therefore, to distinguish between EoE and related clinical disorders such as GERD, both endoscopic and histopathologic tests are required. In the present case, endoscopy findings were consistent with EoE and the diagnosis was confirmed by multiple biopsies.

In adults, physical examination may be necessary to identify any comorbid allergic diseases [1]. In this case, in addition to the patient's advanced age, another notable characteristic was his lack of personal or family history of allergic diseases such as asthma, atopic dermatitis, rhinitis, and food allergies. This observation closely reflects that of others who noted low rates of atopy in elderly patients enrolled in large cohorts [7].

## Conclusions

The present case report emphasizes that EoE may occur in elderly patients. Gastroenterologists should be vigilant for this esophageal disorder in any elderly adult with dysphagia or other symptoms suggestive of EoE. Biopsies should be taken from the proximal and distal esophagus to confirm the correct diagnosis. The patient in this case responded well to eight weeks of PPI therapy using dexlansoprazole and remained symptom-free three months thereafter. In patients with EoE, pharmacologic treatment avoids any unwanted psychological impact of dietary therapy. This might be a concern especially in older adults.
